# Effects of Vacancy Cluster Defects on Electrical and Thermodynamic Properties of Silicon Crystals

**DOI:** 10.1155/2014/863404

**Published:** 2014-01-12

**Authors:** Pei-Hsing Huang, Chi-Ming Lu

**Affiliations:** Department of Mechanical Engineering, National Pingtung University of Science and Technology, Pingtung 912, Taiwan

## Abstract

A first-principle plane-wave pseudopotential method based on the density function theory (DFT) was employed to investigate the effects of vacancy cluster (VC) defects on the band structure and thermoelectric properties of silicon (Si) crystals. Simulation results showed that various VC defects changed the energy band and localized electron density distribution of Si crystals and caused the band gap to decrease with increasing VC size. The results can be ascribed to the formation of a defect level produced by the dangling bonds, floating bonds, or high-strain atoms surrounding the VC defects. The appearance of imaginary frequencies in the phonon spectrum of defective Si crystals indicates that the defect-region structure is dynamically unstable and demonstrates phase changes. The phonon dispersion relation and phonon density of state were also investigated using density functional perturbation theory. The obtained Debye temperature (**θ**_*D*_) for a perfect Si crystal had a minimum value of 448 K at *T* = 42 K and a maximum value of 671 K at the high-temperature limit, which is consistent with the experimental results reported by Flubacher. Moreover, the Debye temperature decreased with increases in the VC size. VC defects had minimal effects on the heat capacity (*C* *_v_*) value when temperatures were below 150 K. As the temperature was higher than 150 K, the heat capacity gradually increased with increasing temperature until it achieved a constant value of 11.8 cal/cell*·*K. The heat capacity significantly decreased as the VC size increased. For a 2 × 2 × 2 superlattice Si crystal containing a hexagonal ring VC (HRVC10), the heat capacity decreased by approximately 17%.

## 1. Introduction

Because of their abundance in the Earth's crust and their unique optical, electrical, magnetic, and catalytic properties, silicon- (Si-)based semiconductor materials have been employed for an increasingly wide range of applications [1–8]. Additionally, with innovative developments in the electronics and microelectromechanical industries as well as light energy conversion devices, Si-related materials have been continually investigated in recent years, and Si-based materials have become the most critical materials for optoelectronic products [1–8]. As the size of electronic components is continually reduced, the effects of size and surface confinement not only lead to changes in thermal conductivity and electron transport properties, but also produce significant variations regarding optical and mechanical properties [9]. The effects of carrier confinement become especially critical when the size of materials reaches the nanoscale.

Perfect crystal materials do not exist in nature, and defects comprising vacancies and interstices occur in various natural materials [9, 10]. Some of these defects are innate, but others are created during the material manufacturing or processing stages. Microscopic structural defects can cause localized electron density changes and redistribution, induce scattering during the carrier (i.e., electron and phonon) transport processes [4], and result in changes in thermal conductivity [5, 6]. These effects reduce the mechanical reliability of the material structure and efficiency of electronic circuits, even shortening the lifecycles of system components. Particularly, more significant effects are induced when microscopic defects occur in nanoscale materials, mainly because carriers in the mesoscopic range possess elastic scattering, whereas those in the macroscopic range demonstrate inelastic scattering. When materials are reduced from a macroscopic three-dimensional structure to a smaller dimensional structure (e.g., zero-dimensional nanoparticles and one-dimensional nanowires), changes in the band structure and the density of states (DOS) near the Fermi energy level occur, and the correlation between the phonon dispersion and phonon group velocity is affected [11, 12]. This generates an energy filtering effect, increases the interface scattering of phonons, and causes alterations in the thermal conductivity coefficients of materials [7].

Recently, various theoretical, numerical, and experimental methods have been employed to investigate the physical properties of Si materials. Dai et al. [2] adopted the lattice kinetic Monte Carlo method to examine the morphological evolution of voids and defects during high-temperature Si crystal growth. Lee et al. [3] combined the Metropolis Monte Carlo method, tight-binding molecular dynamics, and density functional theory (DFT) to investigate interstitial defect growth in crystalline Si. Lysenko and Volz [8] used the scanning probe experimental method to measure the thermal conductivity of porous Si and determined that the thermal conductivity coefficient *k* was significantly smaller than that of bulk single-crystal Si and isotopically pure Si crystals (measured using a steady-state heat flux method). Poter et al. [11] conducted simulations of the phonon dispersion curve and relevant thermal properties of silicon using the Stillinger-Weber, Tersoff, and hybrid potential energy functions. They confirmed that the thermal expansion coefficient, elasticity coefficient, and yield strength values derived from the Stillinger-Weber potential energy function were consistent with experimental values and that the simulated phonon dispersion curve and specific heat approximated those obtained during experiments. Currently, the majority of numerical studies have focused on exploring the properties of perfect Si crystals and nanostructures [12]. However, numerous issues regarding the effects of vacancy cluster (VC) defects on the electrical and thermal properties of Si semiconductor materials require further clarification. Therefore, this study employed first-principle calculations to investigate differences in the electrical and thermodynamic properties between perfect Si crystals and crystals with VC defects. In addition, changes in band structures and DOS were explored, and corresponding relationships between defects and various thermal properties, such as heat capacity (*C*_*v*_), enthalpy, and free energy, were analyzed.

## 2. Numerical Method

Si semiconductor material possessing a diamond structure is part of the Fd3m (No. 227) group and is composed of two superimposed face-centered cubic structures at a distance of (1/4, 1/4, 1/4) *a*_0_, where *a*_0_ represents the Si lattice constant (*a*_0_ = 5.4309 Å). Each unit cell is composed of 8 Si atoms. In this study, a first-principle plane-wave pseudopotential method based on DFT calculations was employed to analyze the electrical and thermodynamic properties of perfect Si crystals and crystals containing VC defects. The following three types of VC defects were explored: (a) a single atomic vacancy (VC_1_), (b) a tetrahedron VC (TVC_5_), and (c) a hexagonal ring VC (HRVC_10_). The subscript numbers in the acronyms represent the number of vacant atomic sites in the crystals (details concerning cluster defect shapes and relevant data are provided in Table 1).

The Cambridge serial total energy package (CASTEP) [13–18] provided by Taiwan's National Center for High-Performance Computing was used to conduct theoretical calculations. First, structural optimization of the total energy for the Si crystal model was performed using the Broyden-Fletcher-Goldfarb-Shanno method [15], which adopts a plane-wave pseudopotential approach to describe the potential energy of electron-ion interactions. The electronic wavefunctions are expanded through a plane-wave basis set to determine the plane-wave cutoff energy and calculate a selected function, and the local-density approximation (LDA) method is used to describe the exchange-correlation potential. As the cutoff energy for calculation increases, numerical error decreases, but computational load substantially increases. In this study, the cutoff energy with a value of 350 eV was selected, at which value of the simulations of total energy and lattice constant were approximately constant. Self-consistent field (SCF) calculations adopt the special *k*-point sampling method of the Monkhorst-Pack scheme [13] to conduct Brillouin-zone (BZ) integration, with a *k*-point mesh of 4 × 4 × 4, a *k*-point interval of 0.5 nm^−1^, and a convergence precision of 10^-6 ^eV/atom. All simulations employed ultrasoft pseudopotentials in reciprocal space during calculations. Finally, density functional perturbation theory (DFPT) was adopted to calculate phonon and thermal properties. Phonon scattering was determined using the norm-conserving pseudopotential method (NCPM) proposed by Hamann et al. [18], where the cutoff energy was 350 eV. The convergence precision of SCF was 10^−6 ^eV/atom, a 2 × 2 × 2 BZ mesh was selected for *k*-point density, the* k*-point interval was 0.07 nm, and a 2 × 2 × 2 supercell was used for the simulation system. This model was employed to calculate band structure, DOS, phonon spectrum, and phonon DOS properties.

## 3. Results and Discussion

**Figure 1:**
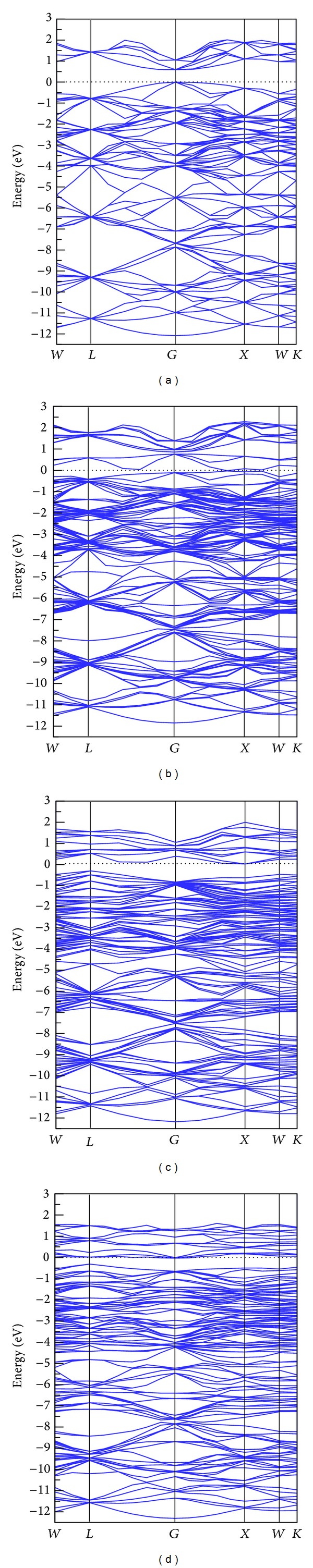
The calculated band structure for (a) perfect, (b) VC_1_, (c) TVC_5_, and (d) HRVC_10_ Si crystals.

Table 1 shows the first-principle calculation results regarding the lattice constants and band gape changes for Si crystals containing VC_1_, TVC_5_, and HRVC_10_ defects after structural optimization. The simulation results indicate that VC defects altered the lattice structure and length of the covalent bonds surrounding the defects. The lattice constant and band gap declined as the VC size increased. Figures 1(a)–1(d) show the band structures along the BZ high-symmetry points for a perfect Si crystal and crystals containing VC_1_, TVC_5_, and HRVC_10_ defects; the corresponding calculation of high-symmetry* k*-point paths in BZ is summarized as shown in Table 2. The dotted line in Figure 1 represents the zero-point energy of the Fermi energy level. The distance between the highest point of the Fermi energy level valence band (*E*_*v*_) and the lowest point of the conduction band (*E*_*c*_) can be used to determine the energy required for valence electrons to move to the conduction band, which is also known as the forbidden band or the band gap. The simulation results in Figures 1(a)–1(d) demonstrate that the band structure ranged between approximately −12.5 and 2.3 eV. The maximum value of the valence band and the minimum value of the conduction band were located on different symmetry points, indicating that forbidden bands for Si crystal are indirect band gaps. Furthermore, the system band gap became narrow as the sizes of VC defects increased, which could be attributed to the increased probability of carrier scattering near the defects. Perfect crystals have a band gap value of 0.59 eV, which differs slightly from the experimental value of 1.1 eV. This result occurs commonly when the generalized gradient approximation and LDA methods are adopted to calculate band gap values [19]. However, this result does not affect energy band and electronic structure analyses. The band structures shown in Figures 1(b)–1(d) indicate that Si crystals with VC_1_, TVC_5_, and HRVC_10_ defects possess band gap values of 0.31, 0.25, and 0.21 eV, respectively. A comparison between the band structures of defective and perfect crystals shows that as the VC defects increased, the distribution areas of the band structures became more concentrated and the band gap decreased. This phenomenon occurred because the VC defects in the crystals altered the distributions of the energy bands and localized electron densities. Due to the increased probability of electron scattering, the electron mobility of Si crystals with VC defects changes, which can affect the semiconductor properties of Si crystals.

**Figure 2:**
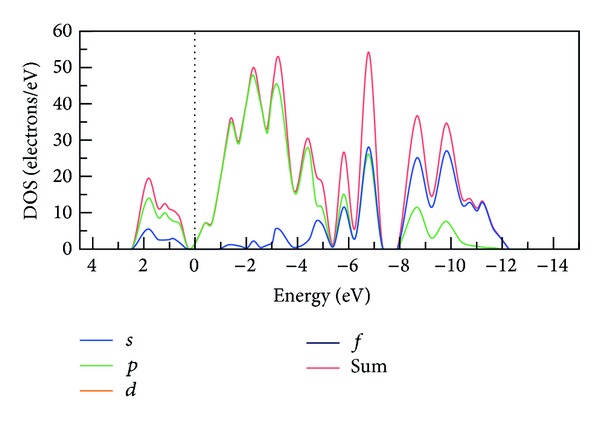
Total DOS and partial DOS of electrons for perfect Si structure; vertical dotted line indicate Fermi line.

Figure 2 shows the corresponding DOS distribution curves for perfect Si crystals under various energy levels. A high DOS at a specific energy level indicates that numerous states are available for occupation, whereas a DOS of zero suggests that no states can be occupied at a given level. The calculation results shown in Figure 2 demonstrate that Si conduction and valence bands are primarily formed by the s- and p-orbitals and have distinct band edges; consequently, band gaps can be clearly defined. Specifically, the conduction band is extended to 2.36 eV and the valence band to −12.27 eV. The corresponding DOS distribution curves for Si crystals with VC_1_, TVC_5_, and HRVC_10_ defects indicate conduction bands extending to 2.15, 2.09, and 1.90 eV and valence bands extending to −12.34, −12.40, and −12.49 eV. Compared to a perfect crystal structure (Figure 2), when the VC size of a defective crystal increased, the DOS of the valence band expanded and the width of the conduction band decreased. In addition, as the VC defect size increased, the DOS curves near the Fermi energy level became relatively smooth, and the corresponding DOS values were comparatively small. An increase in the valence band width indicates that electron delocalization has increased, reducing the band gap. Simultaneously, the narrowing of the conduction band denotes weakened electron delocalization. Therefore, the occurrence of VC defects causes significant changes in the electrical conductivity of Si crystals because the dangling bonds, floating bonds, and high-strain atoms surrounding defects generate a defect level. This level is a type of localized state in which electrons are confined to a certain region of the structure. These excess energy gap states create an area inside the material that causes it to behave similarly to metal.

**Figure 3:**
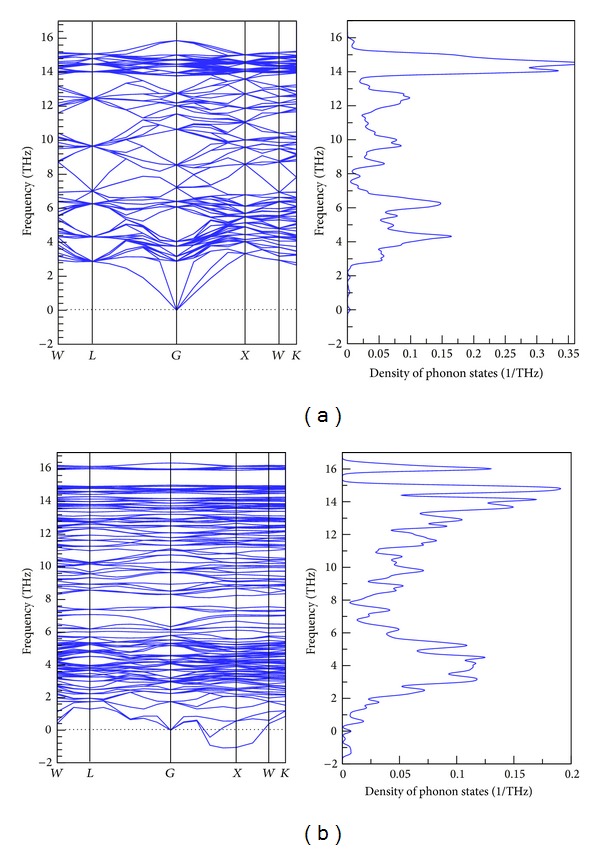
Phonon dispersions and projected phonon density of states for (a) perfect and (b) HRVC_10_ Si crystals.

**Figure 4:**
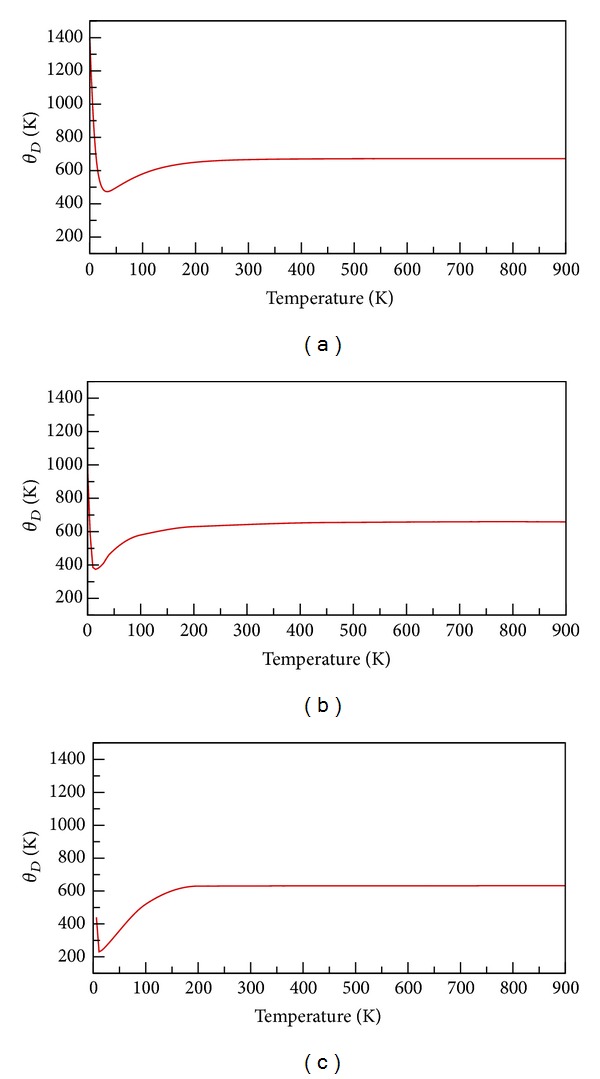
Debye temperature (*θ*_*D*_) of (a) perfect, (b) VC_1_, and (b) HRVC_10_ Si crystals as a function of temperature.

To explore the effects that VC defects have on the thermodynamic properties of Si crystals, CASTEP first-principle calculations and the isovolumetric specific heat calculation method derived by Baroni et al. [16] were employed to analyze thermodynamic properties and temperature effects. The curve distributions in Figures 3(a) and 3(b) demonstrate the phonon dispersion relation and phonon DOS along the BZ high-symmetry points for a perfect crystal and a crystal with an HRVC_10_ defect. Phonon DOS (or vibrational density of states) is calculated by the integration over the Brillouin zone and all 3*N*  phonon bands, where *N*  is the amount of atoms in the cell. The partial (or projected) phonon DOS is obtained by a contribution from the given atom to the total phonon DOS. The contribution to the partial DOS on atom  *i*, from each phonon band, is calculated by the following [15]:

(1) $$\begin{array}{c} N_{i}\left(E\right)=\int \frac{dk}{4\pi^{3}}{\left|e_{j}\left(i\right)\right|}^{2}\delta \left(E-E_{n}\left(k\right)\right), \end{array}$$

where *e*_*j*_ is the eigenvector associated with the mode of energy *E*_*j*_. The partial density of states (PDOS) is then obtained by summation of these contributions over all phonon bands. By construction, all the partial phonon DOS sums up to the true phonon DOS [15]. The left-hand diagram in Figure 3(a) shows that no imaginary frequencies occurred in the phonon dispersion relation of the perfect crystal, indicating that the crystal structure was fairly stable. By contrast, Figure 3(b) shows that a frequency of less than zero appeared in the phonon spectrum for the Si crystal containing an HRVC_10_ defect, which suggests that portions of the crystal structure were unstable and that the structure of the defect regions could cause crystal phase changes or gliding. Moreover, the dispersion relation and phonon DOS shown in Figure 3(b) demonstrate that the HRVC_10_ defect induces two additional band gaps in its vibration modes, that is, 7.55–8.25 THz and 15.01–15.92 THz. Regarding DOS, the high-frequency peak value of DOS for a Si crystal containing an HRVC_10_ defect was lower than that for a perfect crystal, and the reduced portion transferred to 15.92–16.22 THz, forming another peak value and band gap. These results demonstrate that the vacancy defect region possessed a stronger phonon scattering effect. As the size of VC defect increased, the proportion of phonons distributed in low-frequency regions became larger, thereby reducing the slope of the overall dispersion curve and slowing phonon group velocity. This further induced a lower thermal conductivity coefficient for the crystal containing the HRVC_10_ defect when compared to the perfect crystal. Moreover, a material's electronic structure and elastic modulus can be used to estimate its Debye temperature (*θ*_*D*_), which is commonly employed to identify the high- and low-temperature areas of a solid. The value of the Debye temperature at a given temperature is obtained by calculating the actual heat capacity (*C*_*v*_^*D*^) by the following [17]:

(2) $$\begin{array}{c} \theta_{D}\left(T\right)=T{\left[\frac{9Nk}{C_{v}^{D}}\int_{0}^{\omega D/T}\frac{x^{4}e^{x}}{{\left(e^{x}-1\right)}^{2}}dx\right]}^{1/3}, \end{array}$$

where  *N*  is the amount of atoms per cell. When *T* > *θ*_*D*_, all vibration modes have an energy of  *k*_*B*_ *T*; in other words, the heat capacity of the material tends to be a fixed value. However, when *T* < *θ*_*D*_, all high-frequency modes fail and the material's heat capacity decreases as the temperature declines. Therefore, Debye temperature calculations possess critical physical meaning. Figures 4(a)–4(c) show the Debye temperature curve of a perfect Si crystal and crystals with VC_1_ and HRVC_10_ defects at temperatures between 0 and 1000 K. The *θ*_*D*_ value of a perfect Si crystal is 671 K at the high-temperature limit, which is consistent with the experimental results (674 K) reported by Flubacher et al. [20]. Furthermore, the predicted Debye temperature curve has a minimum value of *θ*_*D*_ = 448 K at *T* = 42 K. This result is consistent with the experimental data (i.e., a minimum Debye temperature of 462 K at *T* = 38 K). In addition, because the Si crystal contains vacancy defects, the Debye temperature curve shifts downward and toward the bottom left of the figure, as shown in Figure 4. Particularly, the Debye temperatures of Si crystals with VC_1_ and HRVC_10_ defects, respectively, decrease to 655 K and 632 K at *T* = 800 K. Because the Debye temperature can be used to identify the covalent structural strength of crystals, higher Debye temperatures typically suggest superior mechanical strength and thermodynamic stability. Consequently, the Debye temperature calculation results in Figure 4 are consistent with the analysis results of phonon DOS in Figure 3.

The results of a calculation of phonon spectra can be employed to compute energy (*U*), free energy (*F*), entropy (*S*), and lattice heat capacity (*C*_*v*_) as functions of temperature. The CASTEP total energy yields the total electronic energy at 0 K. The temperature dependence of the energy is calculated by the following [16, 17]:

(3) $$\begin{array}{c} U\left(T\right)=E_{\text{tot}}+E_{\text{zp}}+\int \frac{\hbar \omega }{\exp\left(\hbar \omega /kT\right)-1}F\left(\omega \right)d\omega , \end{array}$$

where *E*_zp_ is the zero-point vibration energy,  *k*  is Boltzmann's constant,  *ℏ*  is Planck's constant, and  *F*(*ω*)  is the phonon DOS. The vibrational contribution to the free energy (*F*) and the entropy (*S*) are expressed as

(4) F(T)=Etot+Ezp+kT∫F(ω)ln[1−exp(−ℏωkT)]dω,S(T)=k{∫ℏω/kTexp(ℏω/kT)−1F(ω)dω−∫F(ω)[1−exp(−ℏωkT)]dω}.

The lattice contribution to the heat capacity, *C*_*v*_, is

(5) $$\begin{array}{c} C_{v}\left(t\right)=k\int \frac{{\left(\hbar \omega /kT\right)}^{2}\exp\left(\hbar \omega /kT\right)}{{\left[\exp\left(\hbar \omega /kT\right)-1\right]}^{2}}F\left(\omega \right)d\omega . \end{array}$$

**Figure 5:**
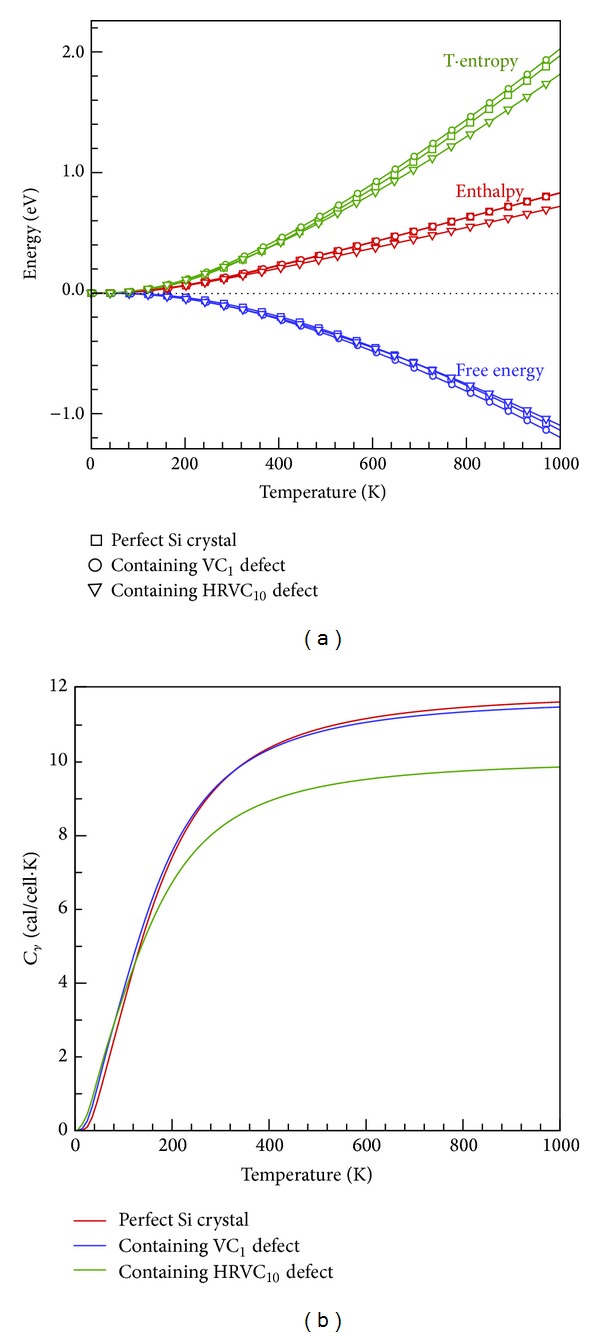
(a) Enthalpy, entropy, and free energy (b) heat capacity for perfect, VC_1_, and HRVC_10_ Si crystals as a function of temperature.

Figure 5(a) shows the correlations between enthalpy, free energy, entropy, and lattice temperature. As temperature increases, the enthalpy and entropy values increase, whereas free energy decreases. This result indicates that increases in the internal energy of the entire system at high temperatures cause disorder in the crystal structure. Furthermore, Figure 5(b) shows the *C*_*v*_ and temperature relationship for a perfect Si crystal and crystals containing VC_1_ and HRVC_10_ defects: when the temperature range was between 0 and 400 K, heat capacity increased dramatically as the temperature increased, and when the temperature exceeded 400 K, changes in heat capacity slowed and gradually approximated a constant (i.e., *C*_*v*_ = 11.85, 11.70, and 9.78 cal/cell·K for perfect, VC_1_, and HRVC_10_ Si crystals, resp.). In addition, Figure 4(b) clearly exhibits that when the temperature was below 150 K, the heat capacity versus temperature curves of the perfect crystal and crystals containing VC defects approximately overlapped, implying that the effect of VC defects on heat capacity was insignificant in low-temperature states. When the temperature exceeded 400 K, heat capacity rapidly decreased with the increasing size of the VC defects. For example, the heat capacity of the HRVC_10_ crystal decreased significantly to 9.78 cal/cell·K at *T* = 1000 K. This result can be attributed to a stronger phonon scattering effect, reduce phonon group velocity, and lower Debye temperatures at the boundaries of the VC defects, which generated a substantial decline in endothermic capacity and a decrease in thermal conductivity for the Si crystal.

## 4. Conclusion

The first-principle plane-wave pseudopotential method was conducted to investigate the effects of VC defects on the electrical and thermodynamic properties of Si crystals. The formation of a defect level produced by dangling bonds and floating bonds surrounding the VC defects caused significant changes in the electrical conductivity of Si crystals. The excess energy gap states create an area inside the defective silicon crystals which causes it to behave somewhat similarly to metals. As the size of VC defect increased, the proportion of phonons distributed in low-frequency regions became larger, thereby reducing the slope of the overall dispersion curve and slowing phonon group velocity. This further induced a lower thermal conductivity coefficient for the crystal. The Debye temperature values obtained in this study consist with the experimental results [20]. As the Si crystal contains vacancy defects, a significant decrease in Debye temperature was observed.

## Figures and Tables

**Table 1:** Patterns of vacancy cluster defect and their corresponding lattice constant and band gap.

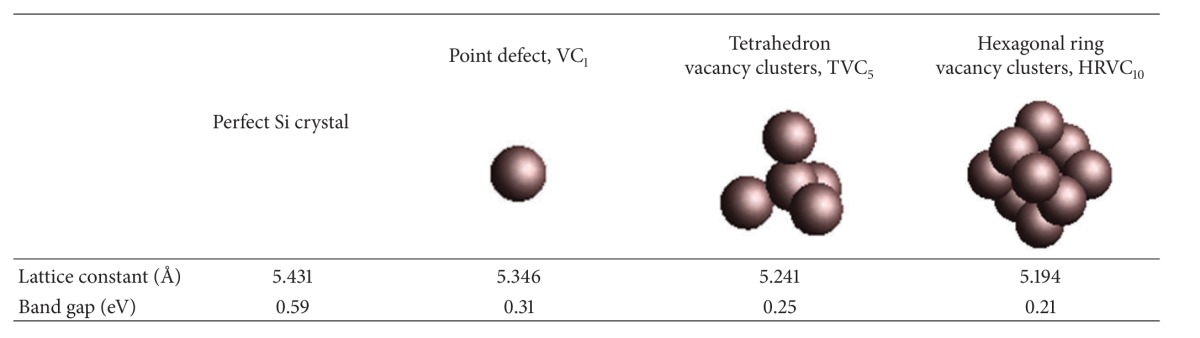

**Table 2:** List of high-symmetry *k*-point paths.

Symbol of symmetry points	Starting points	Corresponding points	Symbol of symmetry points
*W*	(0.5, 0.25, 0.75)	(0.5, 0.5, 0.5)	*L*
*L*	(0.5, 0.5, 0.5)	(0, 0, 0)	*G*(Γ)
*G*(Γ)	(0, 0, 0)	(0.5, 0, 0.5)	*X*
*X*	(0.5, 0, 0.5)	(0.5, 0.25, 0.75)	*W*
*W*	(0.5, 0.25, 0.75)	(0.375, 0.375, 0.75)	*K*
